# Hydration Considerations to Improve the Physical Performance and Health of Firefighters

**DOI:** 10.3390/jfmk9040182

**Published:** 2024-10-02

**Authors:** Angelia M. Holland-Winkler, Blake K. Hamil

**Affiliations:** 1 Department of Kinesiology, Augusta University, 3109 Wrightsboro Road, Augusta, GA 30909, USA; 2 Department of Medicine, Augusta University, 1120 15th Street, Augusta, GA 30912, USA; bhamil@augusta.edu

**Keywords:** dehydration, tactical athletes, hydration assessments, exercise, saliva osmolality, kidney disease, fluid balance

## Abstract

**Background/Objectives**: Firefighters are exposed to a high level of stress as they often perform physically challenging work in hazardous environments while responsible for rescuing and keeping those around them safe. To add to this stress, they are also required to work in heavy, unbreathable personal protective equipment which promotes dehydration. These occupational demands paired with dehydration may lead to increased core temperatures, cardiac strain, and overall risk for sudden cardiac events. Thus, it is important to include hydration assessments and determine fluid needs when firefighters are on shift to ensure their personal safety as well as the safety of those around them by optimizing physical performance by maintaining adequate hydration. Therefore, the purpose of this review is to identify markers of hydration, classifications of hydration status, current hydration recommendations, and hydration interventions that may contribute to the overall clarity of hydration protocols that may optimize performance and health of firefighters. In addition, the impact of common medications, exercise training, and health conditions on hydration status related to firefighters will be discussed. **Methods**: A comprehensive literature search was conducted to discuss the purpose statements. **Results**: Hydration recommendations for firefighters include (1) assessing hydration status with multiple measurements including body mass, urine specific gravity and thirst sensation, and (2) following general hydration recommendations on rest days and exercise hydration protocols during firefighting activities which may be altered according to hydration status measurements. **Conclusion**: Randomized controlled trials in firefighters are needed to determine the impact of maintaining adequate hydration on health markers.

## 1. Introduction

The extreme physical demands associated with firefighting often produce compounding bodily threats to the firefighter, which may lead to immediate or chronic health impairments such as cardiovascular disease (CVD). CVD is common in firefighters, claiming many lives both on and off duty [1]. Current national statistics indicate that sudden cardiac death accounted for the largest proportion of on-duty firefighter deaths from 1977 to 2019 [2,3]. In the past 10 years, 44% of on-duty fatalities have been cardiac-related [2]. Autopsy examinations reveal that 80% of the firefighters whose fatalities were related to cardiac events also had atherosclerotic CVD [4]. Moreover, reports show that there are 17 non-fatal on-duty cardiovascular incidents for every heart disease-related fatality in the US fire service [5]. CVD risk factors that are prevalent in the firefighter population include hypertension, dyslipidemia (specifically, high triglycerides and low HDL cholesterol), obesity, type II diabetes mellitus, and smoking [5]. Daily firefighting occupational stressors such as heat stress and dehydration combined with underlying CVD risk factors may lead to sudden cardiac events [6].

Dehydration is a threat to firefighters as it is difficult to avoid and may result in other serious conditions when paired with heat stress and physical exhaustion. Reports show that over 90% of firefighters are continually dehydrated, causing dehydration to be a major concern in this population [2]. Many firefighters begin their shift in a dehydrated state which is further exacerbated by firefighting events [7]. Firefighters are continually involved in several daily activities that require extra hydration to maintain normal hydration levels, such as low-intensity physical activity over long periods, intense activity, wearing impermeable personal protective equipment (PPE) that weighs 50–60 lb, and continual exposure to hot, smokey environments [2]. Even short-duration firefighting (i.e., fighting a structural fire) and/or firefighting on cool days have been shown to lead to significant dehydration that must be quickly remediated [7,8]. The effects of wearing PPE are especially concerning as its impermeability restricts evaporative cooling which significantly elevates sweat rate that may result in dehydration [2].

Optimal systemic hydration is required to maintain adequate thermoregulatory function [9]. Dehydration, which is the loss of ≥2% of total body water, significantly alters cardiovascular and thermoregulatory function [10]. A loss of body water causes a reduction in plasma volume, which reduces stroke volume, which is the amount of blood pumped from the heart per beat [11,12]. To maintain cardiac output (the amount of blood pumped from the heart per minute) when faced with lower stroke volume from dehydration, the dehydrated state triggers an increase in sympathetic nervous system activity, which leads to an elevated heart rate whereby sustaining cardiac output and mean arterial blood pressure [13]. Thus, during dehydration, cardiovascular strain increases, which leads to a future risk of developing CVD [8]. Suboptimal fluid intake over time leads to a physiological state that, in turn, may lead to the development of various disorders, including hypertension, coronary artery disease, heart failure, type 2 diabetes, and obesity. Endothelial function and arterial stiffness, measures of CVD, are also significantly impaired by dehydration [14,15].

The extreme physical obligations required to complete the occupational duties of a firefighter, such as wearing PPE, carrying heavy loads, climbing, crawling, and all aspects of the various situations they face daily, further challenge the ability of a firefighter to maintain thermoregulatory function, especially in a dehydrated state. The ability to avoid an over-exertional and/or heat-related injury relies on the maintenance of cardiac output, blood pressure, and blood flow to the organs [16,17]. The extent of dehydration can dictate the physiological responses that occur during prolonged physical activity. The greater the level of dehydration, the greater the heart rate response, rectal temperature, and antidiuretic hormone released during and following prolonged exercise [18,19,20].

Intermittent as well as prolonged physical activity in a dehydrated state results in increased systemic vascular resistance and reduced cardiac output, stroke volume, mean arterial pressure, heat dissipation, blood flow to the skin, and sweat response [16,21]. These responses are magnified when exercising in the heat [22]. Firefighters continuously work in a hot environment due to the thermal properties of their PPE. Therefore, the cardiovascular strain involved in the daily tasks of firefighters can be severe and commonly results in cardiac issues, especially when underlying cardiovascular risk factors are present.

To prevent hydration-related health problems, it is important for fire departments to have hydration strategies and evaluation protocols in place. Although departments may have hydration evaluation strategies informally presented, such as a picture of appropriate urine color in the bathroom, it is important to fully understand the hydration needs of this population working in extreme conditions as well as feasible yet valid options to evaluate hydration status. Therefore, the purpose of this review was to identify markers of hydration, classifications of hydration status, current hydration recommendations, and hydration interventions that may contribute to the overall clarity of hydration needs for firefighters.

## 2. Fluid Balance

Water composes the largest percentage of normal body composition, with a range of 50 to 70% depending on age, sex, and body composition [23]. Total body water is distributed into two fluid compartments, intracellular and extracellular fluid. Two-thirds of the total body water makes up the intracellular fluid, and one-third makes up the extracellular fluid [24]. Extracellular fluid is divided between the plasma and interstitial space [25]. Fluid balance in the body is tightly regulated to maintain serum osmolality and preserve physiological function [26].

Terms related to fluid balance and hydration status are defined in Table 1 to provide clarity as hydration status is further discussed.

Derangements in fluid balance in the setting of hypohydration or hyperhydration can result in serious medical conditions. Fluid loss occurs via the kidney, respiratory, and gastrointestinal systems, as well as sweat for thermoregulation [31]. The loss of hypotonic fluid results in the intravascular fluid becoming hypertonic, resulting in intracellular fluid shifting into intravascular space [32]. Through this mechanism, fluid is lost from all compartments, which results in a hypovolemic hyperosmolar state [32,33]. Electrolyte and osmolality derangements are responsible for many of the dangers of hypohydration that will be discussed in the paper.

## 3. Dangers of Dehydration in Firefighters

### 3.1. Fluid Loss on Shift

The occurrence of dehydration in firefighters is primarily initiated by the elevated loss of body fluids that results from several aspects of the job. Fluid loss occurs when the sweat rate increases as the body works to cool itself through evaporative cooling [34]. Multiple activities and occupational requirements individually increase the body’s need for cooling. First, intense and/or prolonged physical exertion is a commonality during a firefighter’s shift. Firefighters perform physical activities including but not limited to carrying ladders, hoses, axes, and other heavy tools, crawling below smoke while navigating the environment, climbing ladders and stairs with tools and possibly a rescued individual, chopping through ceilings with axes, and dragging individuals of all sizes. The physical activity required for successful job performance may be both intense and prolonged, resulting in fluid loss via increased sweat rate and/or persistent sweating [35,36].

In addition to physical exertion, many of these activities are performed in environments of elevated temperatures and humidity due to fires and/or working in summertime weather [34]. To protect the firefighter from fire-associated heat, firefighters wear personal protective equipment. While this protects from burns, it also encapsulates the body whereby trapping physiological heat and preventing evaporative cooling [37,38]. Thus, the sweat rate increases to cool the body, but without success due to restricted airflow inside the gear [39]. Furthermore, most of the calls that firefighters respond to are emergency situations. The psychological response to an emergency may increase physiological stress responses and lead to an increased sweat rate. The combination of these factors, including physical exertion, heat, and stress, leads to a significantly elevated sweat rate and fluid loss, which may cause dehydration.

It is imperative to replenish fluids when dehydrated to prevent starting the next firefighting event in a dehydrated state [40]. However, recovery time and liquid resources to rehydrate may be limited when working at an emergency site for prolonged periods, especially when short-staffed. When working in a dehydrated state, blood volume is reduced which increases heart rate and blood pressure to maintain cardiac output to the brain. Hypohydration occurs when there is a total body water deficit of ≥2% body mass; this hydration status negatively alters thermoregulatory function, thereby compounding physiological heat stress occurring from the many aspects of firefighting (i.e., physical exertion, PPE, environmental temperatures, and stress) [33].

### 3.2. Hydration and Core Temperature

Body temperature sensors are located both centrally in the blood vessels, abdomen, and hypothalamus, as well as peripherally in the skin [9,41]. The hypothalamus receives signals and responds to regulate core and skin temperature [42]. For instance, if the core temperature rises higher than its typical temperature at baseline, an error signal will be sent to the hypothalamus. The hypothalamus will send an effector response to the periphery to possibly increase sweat rate which will lead to evaporative cooling and/or increase cutaneous dilation and thus skin blood flow to promote cooling via dry heat loss [9,41]. The effector response sent from the hypothalamus increases in proportion to the error signal sent to the hypothalamus from the core temperature sensors; this increases the peripheral cooling actions, thereby promoting reductions in core temperature [42]. When skin temperature is warmer than core temperature, sweat rate and vasodilation will increase to provide skin blood flow for evaporative cooling [43,44].

Interstitial fluid and plasma provide most of the fluid lost in sweat, which reduces plasma volume. Most of the sodium and chloride in sweat are reabsorbed into the plasma, which increases plasma osmolality [45]. The increase in osmolality promotes fluid to move from intracellular to extracellular space to replenish the lost plasma volume; however, the ability to return plasma volume to normal will depend on the severity of the water deficit. In addition, plasma hyperosmolarity signals the central nervous system to increase the body’s core temperature sweating threshold, which reduces sweat rate and skin blood flow at a given core temperature [46]. The reduced plasma volume and increase in osmolality that occurs with hypohydration leads to increased core body temperatures of 0.1–0.2 degrees Celsius for every 1% hypohydration [9].

## 4. Hydration Assessments

Blood variables are often considered to be the most reliable form of measuring hydration status, though there is no consensus on which is the gold standard. Blood tests require venipuncture, necessitating a phlebotomist or trained professional, as well as the inherent risk of infection or injury associated with any needle stick. In addition to the added barrier to collecting the sample, analyzing the blood requires the use of advanced laboratory techniques that may be time- and cost-intensive [31,32]. It is important to determine accurate, feasible hydration assessments to implement regularly at fire stations to ensure firefighters are adequately hydrated. The assessments described below may be applicable options for fire stations.

### 4.1. Saliva Osmolality

Salivary osmolality measures the number of osmoles of solute per kilogram of saliva and is indicative of hydration status [47]. Laboratory methods require a trained technician and specialized equipment, including an osmometer. Saliva osmolality has demonstrated accurate changes in hydration status; however, the utilization of this laboratory measure has been difficult to obtain in field settings [7,47,48]. Recently, a portable saliva osmolality meter was designed and validated [49]. Similar to the general population testing blood glucose or ketones from a blood meter, the general population could easily and quickly test their hydration status with this meter from a small amount of saliva. This would serve as a valuable tool for in-field hydration testing for firefighters. As this method is relatively new, more research is needed to determine accuracy during various states of hydration.

### 4.2. Urine

Urine is a source of fluid commonly used to assess hydration. Urine concentration can be measured in three ways: urine specific gravity, urine osmolality, and urine color. Urine-specific gravity and urine osmolality measurements provide accurate numeric data that are easier and less costly than serum testing but still require specialized equipment [27,50]. The use of urine color is the most readily available and uses a Likert scale; as the urine darkens, it suggests a greater degree of hypohydration [51]. While urine is an attractive choice for hydration assessment, it has its own limitations. Its reliability compared to serum osmolality has generated mixed reviews in the literature. Sommerfield et al. compared urine-specific gravity to serum osmolality and found urine to have a high sensitivity but low specificity. This resulted in the classification of euhydrated individuals as hypohydrated via the measure of urine [50]. This can potentially result in the unnecessary removal of firefighters from the line of duty. Overall, urine is an attractive choice for assessing hydration due to its ease of collection and analysis, but it is not without its limitations and is best used in conjunction with another measure. A potential use of urine is demonstrated by the National Athletic Trainers’ Association’s recommendation of using urine color (or specific gravity) along with body mass and thirst sensation for field testing of hydration [33].

### 4.3. Body Mass

Body mass is another tool that can be used to assess hydration. For use in assessing fluid status, body mass must be compared in the acute setting to pre- and post-exertion or to a euhydration baseline [52]. Body mass can be measured via water or air displacement in the laboratory setting, but a floor scale is the most readily available tool of measurement. Researchers have developed equations to control for confounders and account for the intake and output of solids, liquids, and gases that may alter the change in body mass [10]. With energy expenditure and intake controlled for, a gram of body weight loss is equal to one ml of water [27]. Understanding the confounders of body mass measurements is important; however, in the assessment of acute hypohydration of firefighters, a medical-grade floor scale is an acceptable measure when used in conjunction with other hydration measures [33].

### 4.4. Thirst

Thirst is a subjective measure of hydration [53]. Commonly, a Likert scale of 1 to 9, with “1” being “not thirsty at all” and “9” being “very, very thirsty”, is used for assessment. An outcome greater than “3” is commonly associated with hypohydration. Thirst has limitations as a measure both due to its subjective nature and response to hydration status [54]. A suggested pitfall of thirst is the potential for subjects to conceal their true thirst rating in order to be allowed to participate in their goal activity [31]. Additionally, the sensation of thirst is often not recognized until later in the development of hypohydration and is alleviated with rehydration before physiologic euhydration is restored [27]. This makes thirst a low-sensitivity test; however, due to its ease of collection and low cost, it can serve to augment other forms of assessment and help create a robust picture of hydration status [33]. On the other hand, Table 2 provides descriptions of hydration assessments that require more expensive laboratory equipment and may not be as applicable in most fire stations.

In summary, there are many options to choose from when evaluating hydration status. While serological studies such as serum osmolality serve as a de facto gold standard in the laboratory setting, they may be impractical in the field setting. The use of more than one test helps create reliability while still being practical for real-world use. Several suggestions appear in the literature; Cheuvront. et al. suggest the combination of a first-morning body mass weight and urine concentration (measured either through urine specific gravity or color), while the National Association of Athletic Trainers builds upon this approach with the addition of thirst sensation [27,33].

## 5. Hydration Status and Physical Performance

As discussed, firefighters operate in environments of extreme heat and both prolonged and strenuous physical activity, placing them at risk for hypohydration. With the risks of hypohydration previously explained, it is clear to see the importance of maintaining an adequate level of hydration. Not only are the health risks of dehydration an immediate concern of inadequate hydration but suboptimal hydration also leads to decreased physical and cognitive performance [64]. Firefighters’ occupation often places them in harm’s way, and a decrease in physical or cognitive performance could result in serious injury or death.

Armstrong et al. investigated the impact of dehydration and endurance performance in track athletes during 1500, 5000, and 10,000 m races. It was found that all three distances were impacted to a statistically significant degree, with a larger effect size occurring in the longer two distances [65]. Additionally, ambient temperature can impact endurance performance. Of concern to firefighters, temperatures greater than 31 degrees Celsius (87.8 degrees Fahrenheit), were associated with decreased endurance performance [66]. With an unavoidable increase in ambient temperature during firefighting, optimal hydration is of paramount importance to prevent further decreases in the endurance capacity firefighters rely on.

Additionally, strength performance is impacted by hypohydration. A study compared the performance during resistance exercise of athletes in one of three groups, including a euhydrated group, hypohydration of −2.5% of body mass (HY25) group, and hypohydration of −5.0% of body mass (HY50) group. While HY25 and HY50 did not have a decrease in single, maximal strength, cumulative total work completed was decreased compared to the euhydrated group in both hypohydration groups during sets of back squats [67]. This is also of particular importance for firefighters who carry not only personal gear of considerable weight but also other firefighters and victims in emergency situations. Furthermore, hypohydration can impact cognitive performance. The current literature has mixed results on the impact of hydration and cognition [68]. However, results demonstrate a decrease in vigilance and working memory among male participants with acute dehydration of 1.59% change in body mass [69]. Additionally, similar results were observed among females when comparing dehydrated and hydrated states with mood and cognition.

### 5.1. Exercise Fluid Loss and Rehydration

The next consideration in hydration is the loss of fluid that will occur during firefighting physical activities and the ability to rehydrate during and after those activities. Sweating is the primary mechanism by which the body can dissipate heat to the environment [70,71]. Primary factors influencing this sweat loss are environment, physical activity characteristics, and individual variance [64,72]. The level of physical activity has a direct influence on sweat loss; total body fluid loss from exercise has a positive relationship with intensity and duration of exercise [73]. Exercise intensity creates larger thermal stress from increased body metabolism, and as exercise intensity increases, total body sweat does as well [74]. The environment also impacts fluid loss. At low ambient temperatures, the body can expel thermal heat through conduction, radiation, and evaporation due to a positive gradient from the body to the environment. As ambient temperature rises to near that of the body, the gradient is reduced, and evaporation becomes the main source of heat elimination [75]. Thus, elevations in external temperature increase sweat loss which promotes evaporative cooling to prevent internal heat accumulation [76].

Clothing and protective gear add an additional barrier to cooling potential and exacerbate total body sweat loss. The Toronto Fire Department investigated the difference in pants or shorts uniforms for firefighters, and supported the switch to shorts due to a 10 to 15% increase in exercise tolerance time [34]. Finally, individual factors also influence sweat loss among participants during the same activity. Factors such as heat acclimation, fitness, and body weight can influence sweat rates. Because of the many variables and personal variance, it is recommended that firefighters create an individualized hydration strategy by assessing their hydration loss during physical activity [77]. This can be achieved by comparing pre-and post-activity body mass. To do this, several basic assumptions must be made that the change in body mass is equal to total body sweat loss; this includes that respiratory fluid loss and energy expenditure are negligible and can be excluded from the calculation [73]. As a result, the following equation can be applied:

Fluid Loss = Δ body mass − (fluid intake − urine output) − (food intake − solid excretion)

**Figure 1:**
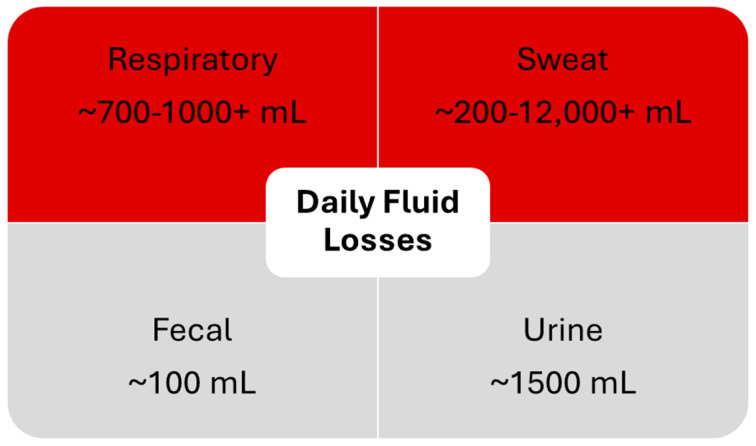
Average daily fluid loss from respiratory, sweat, fecal, and urine fluid. Respiratory and sweat fluid loss are the most variable fluid loss sources, especially in firefighters.

However, respiratory fluid loss is quite variable and may not be negligible as this equation assumes, especially when firefighters are responding to emergencies. Firefighters who work in higher altitudes also lose more respiratory fluid. Figure 1 demonstrates areas of fluid loss, with respiratory and sweat fluid loss being the most variable [78,79,80].

**Figure 2:**
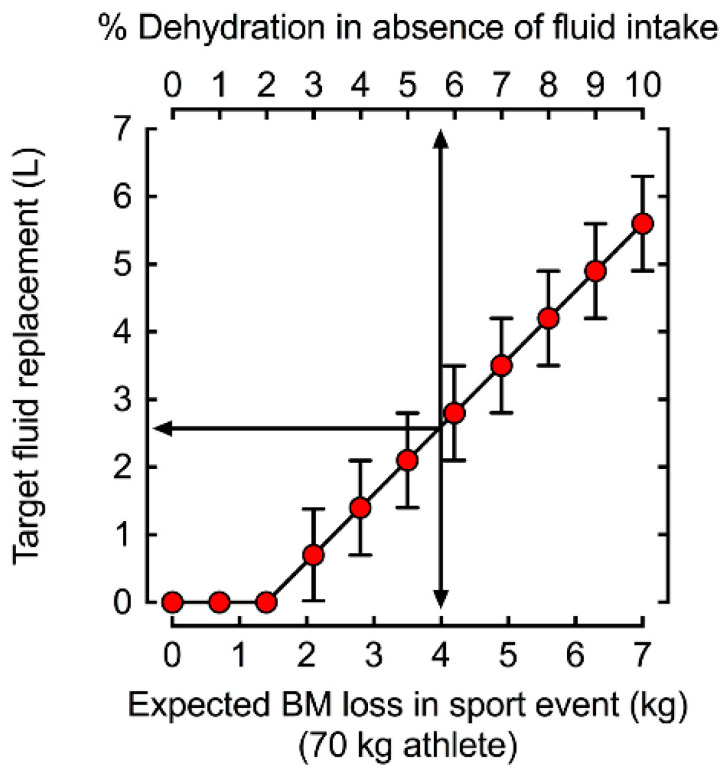
Target fluid replacement estimates based on body mass loss for a 70 kg athlete during exercise to prevent dehydration. Reprinted from “*Practical Hydration Solutions for Sports*”, by L.N. Belval et al., 2019, Nutrients, 11, 1550 [64].

Identifying fluid losses helps determine an appropriate rehydration strategy. In the acute setting, rehydration with a volume greater than the volume lost is recommended due to the expected volume loss to urine and sweat production in the recovery period. Consumption of 150% of the fluid volume lost, or 1.5 L for every kg change in body mass is recommended for rehydration [81,82]. An example of fluid replacement for a 70 kg athlete based on body mass loss is demonstrated in Figure 2 [64].

### 5.2. Electrolyte Loss and Replenishment

Another consideration in the rehydration phase is replenishing lost electrolytes. Electrolytes include sodium, potassium, calcium, magnesium, and chloride, each of which play a role in muscle and nerve function [83]. Electrolytes are lost via sweat; sodium and chloride are the most abundantly secreted [84]. Due to the occupational demands that increase sweating and total daily sweat volume, as previously mentioned, it is imperative that firefighters replenish electrolytes with a focus on sodium. Replenishing sodium provides a greater retention of the fluids consumed for rehydration [81]. However, individual sodium excretion is highly variable. A study quantifying sodium sweat concentration among participants during the summer and winter from different body locations demonstrated both interparticipant variance and intraparticipant variance in the location of sweat and testing setting [78]. With this in mind, it is clear that salt loss is individualized and an important aspect of rehydration.

Typical recommendations for replenishing sodium during rehydration include sports drinks and salt from food without the further need for salt tablets [72,77]. If firefighters are not consuming sodium-rich diets, it would be beneficial to hydrate with an electrolyte solution before starting firefighting activities, during activities that last longer than an hour, and after the activity, in addition to consuming plain water [85,86]. Maintaining electrolyte balance will prevent performance and cognitive decrements, promote better recovery from exhausting activity, and prevent conditions such as hyponatremia [33].

## 6. Experimental Hydration Interventions

Evidence suggests that plasma hypovolemia and hypertonicity that occur from fluid deficits promoted by firefighting occupational demands, such as working in heavy, unbreathable personal protective equipment in hot temperatures, negatively impact the health of the cardiovascular, renal, and other organ systems [87]. Randomized controlled trials demonstrating the impact of hydration on health markers exist but are limited. Association studies demonstrate that higher levels of drinking plain water are significantly associated with a lower prevalence of chronic kidney disease, whereas lower levels of drinking plain water are associated with increased prevalence. Borghi et al. examined the effectiveness of water intake on idiopathic calcium stone disease over five years in a randomized controlled trial with 199 patients after their first idiopathic calcium stone episode and 101 control participants [88]. They found that low urine volume is a risk factor in nephrolithiasis and that drinking large amounts of water reduced the recurrences of kidney stones.

Vasopressin is an antidiuretic hormone produced by the hypothalamus that functions to maintain plasma osmolality by controlling water excretion from the kidneys as well as promoting thirst [89]. Thus, vasopressin is valuable in maintaining fluid balance. Studies have shown that individuals consuming low levels of water have high concentrations of circulating vasopressin. Plasma copeptin is considered a surrogate marker of plasma vasopressin as it is produced in a one-to-one ratio with vasopressin and is more stable than vasopressin [90]. In a six-week trial, Lemetais et al. found that increasing daily water intake over a six-week period reduced circulating copeptin levels in healthy individuals [91].

Increased levels of copeptin have been shown to be significantly related to increased risk for chronic kidney disease in the general population [92]. Thus, increasing daily water intake may help to reduce this risk. A randomized controlled trial demonstrated that it is feasible and safe for patients with stage 3 chronic kidney disease to increase water intake by 0.7 L per day; however, the impact of water on this stage of the disease is yet to be determined [93]. Enhörning et al. conducted a randomized controlled trial to determine if an increase of 3 L per day of water for one week would alter plasma copeptin levels. They also examined an acute intake of one liter of water on copeptin levels over a four-hour period following the water intake. After the acute water intake, copeptin levels were significantly reduced in 30 min and reached a maximal reduction at 90 min which remained low for the rest of the four-hour period. After the one-week period, high water intake was associated with a 15% reduction in copeptin levels, with the greatest reduction occurring in participants who had habitually high copeptin levels [94].

The implications of these hydration interventions on kidney health provide value to the firefighting population as they are at a higher risk for kidney injury and disease due to heat stress and dehydration [95]. In addition, firefighters are at a significantly higher risk for kidney cancer as early as the second decade of their career [96]. Thus, due to the benefits found for increasing plain water consumption on copeptin levels, increasing water consumption throughout the day seems a promising preventative strategy to reduce the risk of kidney complications associated with firefighting.

## 7. Medications and Fluid Balance

The occupational burden experienced by firefighters not only fails to mitigate common chronic health conditions but, in some cases, may contribute to the higher prevalence of disease in comparison to the general population. A cross-sectional study discovered the prevalence of obesity cholesterol, and elevated blood pressure among firefighters was greater than Healthy People 2010 targets. Furthermore, the prevalence of obesity, high low-density lipoprotein, and high total cholesterol was greater than the general population [97]. As such, firefighters are likely to be treated and taking medication to manage these conditions. Given the rigorous demands placed on firefighters, they may be at increased risk of encountering adverse events from medical management. Additionally, medical management may have an impact on hydration status.

More than 38 million Americans have diabetes and 38% of the population has prediabetes [98]. With such a high national prevalence, firefighters taking diabetic medication is not only common now but is likely to increase in the future. Metformin is the most common first-line medication in type two diabetes mellitus. A rare but serious adverse effect of metformin is metformin-associated lactic acidosis (MALA). In MALA, supratherapeutic levels of metformin cause an increase in lactate production with a reduction in lactate clearance, leading to anion gap acidosis [99]. Acute illness, renal insufficiency, and dehydration are common precipitating factors for metformin-associated lactic acidosis [100]. This is a common consideration for elderly patients and those with acute illness, but firefighters’ unique occupational demands warrant consideration as well. Firefighters are susceptible to dehydration and rhabdomyolysis, causing acute kidney injury, which could result in supratherapeutic levels of metformin precipitating MALA. Further, the extreme demands of responding to fires may also require intensive anaerobic exercise, which may worsen metabolic lactic acidosis. Firefighters’ occupational demands are not a contraindication to taking metformin but should be taken into consideration by clinicians when prescribing medication.

The percentage of firefighters with hypertension surpasses that of the general population. Common first-line antihypertensives are angiotensin receptor blockers (ARB), angiotensin-converting enzyme inhibitors (ACE), calcium channel blockers, and diuretics. Diuretics are a versatile medication used in the treatment of not only hypertension, but also heart failure, chronic kidney disease, and liver failure. Diuretics increase free water excretion through urine which presents a clear potential to worsen hypovolemic states. Thiazide diuretics commonly used in hypertension treatments can also lead to electrolyte derangements of hyponatremia, hypokalemia, and hypomagnesemia [101]. ACE inhibitors and ARBs both block the rennin-angiotensin aldosterone system, which blunts increased vascular tone, systemic vascular resistance, and water reabsorption [102]. Hypotension, hypohydration, and metabolite disorders are adverse reactions for firefighters who are at an increased baseline risk for these conditions during emergency response situations. Calcium channel blockers and beta blockers cause vasodilation and decreased cardiac output, respectively [103,104]. Both vasoconstriction and increasing cardiac output are physiological responses to exercise and blockade may be dangerous during high exertional demands [105]. All these medications play a crucial role in managing hypertension. However, because of their propensity to impact fluid status, electrolyte balance and autonomic response, it is imperative to take firefighter’s rigorous occupational demands into account when prescribing.

## 8. Practical Applications and New Techniques

The Institute of Medicine suggests that daily fluid intakes of 3.7 L for men and 2.7 L for women would replenish minimal water losses due to metabolic processes related to minimal conditions (i.e., temperate climate, minimal physical activity, and sweating). However, high altitudes, hot/cold temperatures, increased exercise intensity and/or duration, and elevated sweat rate will increase the daily fluid intake needed to replenish fluid losses. Under extreme conditions, total water losses may reach and/or exceed 8.5 L per day. Firefighters work in those extreme conditions, as previously mentioned. The current literature has no common consensus regarding achieving and maintaining optimal hydration. There is a commonality among the literature that achieving euhydration requires assessing hydration status pre-activity, appropriately evaluating water and electrolyte loss, and accessing fluids for rehydration [64,77].

The American College of Sports Medicine (ACSM) defines the goal of pre-hydration, which is to start the physical activity euhydrated and with normal plasma electrolyte levels [72]. To ensure firefighters achieve euhydration in preparation for their activity, their hydration level needs to be assessed. Assessing hydration can be difficult, but reliable body mass measurements and urine color can give important insights into current hydration status [33]. The use of body mass as an assessment tool relies on daily measurements, and it is imperative that firefighters, using this technique, develop a consistent schedule for body mass measurement, ideally in the morning or pre-shift. A study investigating the fluctuation of morning body weight measurements revealed that three days of consecutive measurements could provide an accurate baseline value, and at nine days, the precision was further improved [63,106]. This baseline is important for ensuring firefighters are adequately hydrated and prepared for their day’s demands and as a reference for post-activity rehydration.

### 8.1. Hydration Protocol for Firefighting Activity

ACSM provides a hydration protocol that should be implemented before, during, and after exercise (i.e., firefighting activity) to start the firefighting activity in a euhydrated state and then replace fluid losses associated with the increased activity and sweat rate [72].

#### 8.1.1. Before Firefighting Activity

It is suggested to start consuming 2 to 3 mL fluid per lb of body mass at least 4 h before the activity. Therefore, a 150 lb person would drink 300 to 450 mL of fluid before the firefighting activity, which could be water and/or a sports drink with electrolytes. This allows time for the fluids to become physiologically useful, with the excess excreted via urine output. If possible, conduct a weigh in before starting the activity and record a starting body mass [72,107].

#### 8.1.2. During Firefighting Activity

Firefighters often cycle on and off during firefighting activity to prevent heat illness. During a break, firefighters should consume water and/or sports drinks to limit dehydration to <2% body mass loss. In addition, cold fluid and popsicles would be beneficial to reduce core temperature often elevated by the PPE, environment, and firefighting activities [72,107].

#### 8.1.3. After Firefighting Activity

After the firefighting activity, conduct another weigh in to determine fluid loss based on the reduction in body mass. It is important to measure body mass wearing the same dry clothes as before the firefighting activity, as wet clothes are heavier. Firefighters should consume 450–675 mL of fluid for every 1 lb of body mass lost via water and sports drinks. Consuming a salty snack or meal would also be helpful to replenish electrolyte losses [72,107].

### 8.2. Heat Acclimation Techniques

In addition to implementing appropriate hydration testing and protocols, acclimating to the heat may reduce the physiological strain associated with firefighting, which may slow the dehydration process [108]. Adaptations that may occur with heat acclimation include increased sweat sensitivity and output, increased blood flow and volume, reduced core temperature and cardiovascular strain, and improved fluid balance [109,110].

Various approaches have been utilized to produce adaptations associated with heat acclimation. Passive heat acclimation occurs without exercise in prolonged warm or hot temperatures, while active heat acclimation combines heat exposure and exercise to induce adaptations [111]. Active heat acclimation occurs naturally in firefighters as they conduct physically taxing work in hot temperatures while wearing PPE. Thus, it may benefit them to focus on implementing passive heat exposure options; however, this still needs to be explored. It is suggested that passive heat acclimation bouts occur for at least 30 min across a minimum of 6 to 7 consecutive days [112]. For instance, sauna bathing is a passive approach used to increase plasma and red-cell volume possibly. Scoon et al. examined three weeks of sauna bathing post-endurance workout on endurance running performance and plasma adaptations in six male competitive endurance runners and triathletes [113]. They found that 30 min of 90 °C sauna bathing for three weeks after endurance training increased plasma volume and improved endurance running performance.

Similar to a hot sauna, hot water immersion is also used as a passive approach to induce heat acclimation. Zurawlew et al. examined daily hot water immersion after endurance exercise on heat acclimation and endurance performance in 17 non-heat acclimatized males [114]. They found that 40 min of 40 °C hot water immersion after exercise was an effective heat acclimation strategy. It reduced rectal temperature after complete submaximal exercise in temperate (18 °C) and hot (33 °C) room temperatures. Also, skin temperature at sweating onset was lower during submaximal exercise in temperate and hot room temperatures after six days of hot water immersion. These adaptations are beneficial to maintaining fluid balance and slowing time to dehydration when working in the heat.

## 9. Conclusions

The physical exertion and environmental heat experienced by firefighters while working in heavy, restrictive personal protective equipment results in elevated sweat output and consequential dehydration. Thus, the development of individualized and feasible hydration strategies is of the utmost importance when determining methods to improve the health and performance of firefighters. The following suggestions and protocols should be taken into consideration when developing a thorough hydration strategy:

- Implement multiple measurements to determine hydration status. A feasible combination suggested by the National Association of Athletic Trainers includes measuring first-morning body mass, urine concentration through specific gravity or color, and thirst sensation.
- On rest days with minimal sweat and respiratory rates, daily fluid intakes of 3.7 L and 2.7 L for men and women, respectively, are suggested. However, fluid intake requirements will increase when sweat and/or respiratory rates increase due to working at higher altitudes, hot/cold temperatures, increased physiological workloads, and/or wearing personal protective equipment.
- The following hydration protocol from ACSM could be utilized to maintain euhydration and prevent heat illnesses from firefighting activities: (1) start consuming 2 to 3 mL fluid per lb of body mass at least 4 h before the firefighting activity, (2) during a break, consume water and/or sports drinks to limit dehydration to <2% body mass loss, and (3) consume 450 to 675 mL of fluid for every 1 lb of body mass lost via water and sports drinks.

Randomized controlled trials demonstrating the effects of maintained euhydration on health markers are limited in populations outside of firefighters and non-existent in firefighters. Firefighters are at high risk for cardiovascular disease, kidney disease, cancer, and metabolic syndrome; thus, future studies must include randomized controlled trials to determine the impact of maintaining adequate hydration on health markers related to these conditions.

## Figures and Tables

**Table 1:** Terminology related to fluid balance and hydration status.

Term	Definition
Hypohydration/ Dehydration (used interchangeably for this review)	>2% of body mass loss from water deficits [10,22]
Euhydration	Normal total body water that fluctuates narrowly [27].
Hypotonic Fluid	Contains a lower concentration of solute compared to plasma and interstitial fluid [28].
Hypertonic Fluid	Contains a higher concentration of solute compared to plasma and interstitial fluid [28].
Serum osmolality	The sum of the osmolalities of every single dissolved particle in the blood, such as sodium and associated anions, potassium, glucose, and urea [28].
Hypoosmolar serum	Serum with a lower concentration of dissolved particles per volume of serum than normal (275 to 295 mOsm/kg) [29,30].
Hyperosmolar serum	Serum with a higher concentration of dissolved particles per volume of serum than normal (275 to 295 mOsm/kg) [29,30].

**Table 2:** Hydration assessments that require laboratory equipment.

Hydration Assessment	Description
Serum Osmolality [27,29,55,56,57]	As fluid is lost in the form of sweat, blood solute concentration will increase, resulting in increased serum osmolality. Normal serum osmolality ranges from 275 to 295 mOsm/kg. Under ideal conditions, osmolality increases by approximately 5 mOsm/kg for a 2% loss of body mass by sweat. Plasma osmolality’s sensitivity in detecting mild hydration deficits is controversial, and it may return to baseline after activity even without hydration.
Blood Hematocrit and Hemoglobin [31,58]	Lowered plasma volume from dehydration creates a higher concentration of red blood cells. This measure provides an analysis of blood plasma volume assuming the individual has a normal total red blood cell count This measure cannot assess intracellular fluid content; thus, it accurately measures changes in plasma volume but not total body water.
Hormones [59,60,61]	Hormones released in response to changes in body fluid status can also serve as a biomarker for hydration. Aldosterone (ALD) and Arginine Vasopressin (AVP) have both been described as markers for hypohydration. High-intensity exercise results in a rise of ALD independent of hypohydration, while AVP rises more with the higher intensity exercise than with hypohydration alone. These effect modifications make ALD and AVP unreliable measures of hypohydration when used alone.
Total Body Water (TBW) [27,31,62,63]	TBW is calculated through the input of an isotope, usually deuterium oxide, (2H2O), and later sampling of bodily fluid to determine the concentration of the isotope. Using this concentration, calculations can be made to determine the total body water; as water content increases, the concentration of the isotope will decrease. Total error in estimating TBW using tracer dilution is reported as low as 1%, allowing for the detection of small changes in fluid status. Despite its accuracy, the technical, equipment, and time requirements for the test make it impractical for many settings, especially when assessing acute changes in hydration status.
